# Establishment of a standard seed lot system of an Iranian Mumps virus strain; RS-12, for mass production of mumps and MMR vaccines

**Published:** 2012-09-30

**Authors:** M K Shahkarami, T Mokhtari Azad, K Aghaiypour, A Shafyi, M Taqavian, A Mohammadi

**Affiliations:** 1 Human Viral Vaccines Department, Razi Vaccine and Serum Research Institute, karaj, Iran; 2 Head of Virology Department, School of Public Health, Tehran University of Medical Sciences, Tehran, Iran; 3 Head of Genomics Lab., Biotechnology Department, Razi Vaccine and Serum Research Institute, Karaj, Iran; 4 Member of scientific board, Human Viral Vaccines Department, Razi Vaccine and Serum Research Institute, Karaj, Iran; 5 Head of Human Viral Vaccines Department, Razi Vaccine and Serum Research Institute, Karaj, Iran

**Keywords:** RS-12, MMR, mumps, vaccine

## Abstract

**Background:**

At present the mumps virus strain used for production of mumps vaccine for our local use is Hoshino strain. However, according to our National public health policies, this strain should be replaced with a safer strain. Based on our previous data, the Iranian mumps strain; RS-12 has been proved to be the most suitable alternative to Hoshino strain with little or no adverse events following vaccination

**Methods:**

The aim of the present study was to optimize propagation of RS-12 strain and prepare standard seeds for vaccine mass production.

The virus was inoculated to cells using different methods and different multiplicity of infection (MOI). The viral suspensions were harvested using different methods. Quality control tests were run at different stages.

**Results:**

Maximum viral yield was achieved when cell suspensions were inoculated at MOI of 1:10 and incubated at 36-37ºC for 48 hours, followed by replacement of the media and incubation at 33-34 ºC for 5-7 days. Filtration did not affect the viral titre. A standard seed lot system was successfully established and experimental batches of MMR vaccines were produced.

**Conclusion:**

The established seed lot system has met all requirements of WHO regulations and could be used in mass production of safe and efficacious mumps and MMR vaccines. Clinical trials are in progress for this newly produced vaccine.

## Introduction

Mumps virus (MuV) belongs to family Paramyxoviridae, subfamily Paramyxovirinae, genus Rubella virus. MuV has a single stranded, non-segmented, negative strand genome; (1). Although only one serotype of MuV has been described; (2), genetic variations exist among MuV strains. Different isolates of MuV have been placed into twelve, A to L, genotypes based on the nucleotide sequence of small hydrophobic (SH) gene which is the most variable gene among MuV genome; (3). MuV could be stable for 3-4 days at room temperature, up to weeks at 4ºC, and years at -50 ºC or below. However, presence of substances such as bovine albumin, gelatine or serum stabilizes the virus; (4).

Natural mumps infection in human is initiated by droplet spread. The most common sites of virus spread are the parotid glands, central nervous system, gonads, kidneys, pancreas, heart ad joints which leads to inflammation in these tissues; (1). Infection is subclinical overall in one quarter to one third of cases; (4, 5) but up to 10% of patients develop aseptic meningitis; a less common but more serious complication is encephalitis, which may cause death or disability; and permanent deafness, orchitis and pancreatitis are other untoward effects; (6).

The first live attenuated mumps vaccine was developed during 1960s; (7) and since then all mumps vaccines in use contains live attenuated viruses; (1).

Adverse reactions following vaccination are major concerns. In addition to allergic reaction to egg protein; (7) or gelatine; (8) serious complications such as aseptic meningitis; (7, 9) may occur in vaccines. The rates of this complication vary according to the vaccine strain, the manufacturer, the case definition, the study design and the intensity of surveillance; (10).

In Iran, mumps and MMR vaccines are manufactured for decades at Razi vaccine and serum research institute. These vaccines contain Hoshino strain for mumps, which as other strains may cause some adverse reactions. According to the recommendations of the Iranian ministry of health, this strain should be replaced with a safer strain. Thus the objective of this study was to design and establish a standard seed lot system of Iranian mumps strain (RS-12) for future MMR vaccine production.

## Materials and Methods

### Mumps virus, Cell substrates and Medium:

Attenuated RS-12 strain of mumps virus; (11) was provided by Human Viral Vaccines Department., Razi Institute, IR Iran.

Human diploid cells (MRC-5) were prepared both as monolayer and cell suspension in disposable flasks as cell substrate for propagation of RS-12 virus. Vero cell monolayer was prepared in sterile glass tubes for titration of harvested viruses. The cells were provided by Human Viral Vaccines Dept., Razi Institute, Iran.

DMEM was used in all stages of cell preparation and virus propagation process.

### Propagation of the virus using MRC-5 cell suspension

To prepare cell suspension, 10 flasks containing MRC-5 cell monolayer were inspected macroscopically and microscopically for contamination and confluency of the cell monolayer, respectively. Media was removed and monolayers were washed with fresh media, and trypsinized. Four hundred ml media (plus 8% bovine serum) was added to each flask and after pipetting, content of each flask was dispensed in 4 flasks.

Since the MOI is defined as the proportion of the number of viral particles per cell, it is necessary to count Cells in the prepared suspension. So, haemocytometer slides were used. After dispensing the suspension in new flasks, the number of cells in each flask was determined according to the volume of suspension in the flask. Normally, each 175 mm^3^ flask containing a confluent monolayer of MRC-5 consist of about 7*10^6^ cells.

40 flasks were divided into 10 groups, including a control group with no virus inoculation. All the remaining flasks were inoculated with the appropriate titre of mumps virus. According to the titre of virus in the seed the flasks were inoculated with the mumps virus at MOI of 1:1, 1:2, 1:5, 1:7, 1:10, 1:12, 1:15, 1:20 and 1:25, sequentially.

The inoculated flasks were incubated at 37ºC for 48 hours. Later, the media was replaced with fresh and serum-free DMEM media and incubated at 33-34ºC.

After appearance of 70% CPE, the flasks were kept overnight at 4ºC. The flasks were shaked thoroughly and the suspension was mixed with an equal volume of stabilizer. Half of the harvested viruses of each group were filtered using a 0.22µm Millipore. Samples were taken from each group of harvested viruses and all of prepared viral suspensions and samples were kept at -40ºC immediately.

### Propagation of the virus using MRC-5 cell monolayers

10 flasks containing MRC-5 cell monolayer were inspected macroscopically for any contamination and microscopically for confluency of the cell monolayer. Their media were removed and monolayers were washed with fresh media. Monolayers were trypsinized and 400 ml media (plus 8% bovine serum) was added to each flask. After pipetting, content of each flask was dispensed into 4 flasks and kept at 37ºC for 6-7 days till confluent monolayers appeared. The media were replaced with fresh and serum-free media. The flasks were kept at 33-34ºC for 48 hours.

40 flasks were divided to 10 groups. One group was left as control with no virus inoculation. The rest flasks were inoculated. According to the titre of virus in the seed, the flasks were inoculated with the mumps virus at MOI of 1:1, 1:2, 1:5, 1:7, 1:10, 1:12, 1:15, 1:20 and 1:25, sequentially. All the flasks were incubated at 33-34ºC.

After appearance of 70% CPE, the flasks were incubated at 4ºC, overnight. The flasks were shaked thoroughly and the suspension was mixed with an equal volume of stabilizer. Half of the harvested viruses of each group were filtered using a 0.22 µm Millipore. Samples were taken from each group of harvested viruses and all of prepared viral suspensions and samples were kept at -40ºC immediately.

### Quality control tests

At least two main quality control tests were performed on each sample; sterility test and virus titration.

Both microbial and mycoplasma contamination were considered as determinants of sterility. Microbial contamination was evaluated by inoculation of each sample to Thioglycolate and soybean casein digest media. Two sets of each media were inoculated; one set was kept at room temperature and the other at 37ºC. The results were read after 14 days. The mycoplasma contamination tests were done by the Quality Control Department, Razi Institute, Iran. PPLO broth and agar were used and the results confirmed by PCR using a general primer for mycoplasma species.

CCID50 method and Karber formula were used for determining the titre of mumps virus in each sample. Macrotitration method in tubes was employed. Vero cell monolayers were prepared in glass tubes. To determine the titre of virus in each sample, 10-fold serial dilutions of the sample were prepared. Each dilution was inoculated to at least 4 tubes (0.1 ml/tube). After 10 days the titre of virus in each sample was calculated using Karber formula as below; (12).

Log CCID_50_ = L - d (S - 0.5)

L: log lowest dilution

d: difference between log dilution steps

S: sum of the proportions of "positive" cell culture tubes.

## Results

During optimization of the cultivation of RS-12 strain, each experiment was repeated 5 times and the data analysed. The mean titre of harvested viruses in each defined condition is summarized in Table 1.

**Table 1:** Mean titre of propagated viruses in different conditions.

		MOI								
		1:1	1:2	1:5	1:7	1:10	1:12	1:15	1:20	1:25
**Susp.**	F	2.15	3.07	3.50	5.85	**6.60**	6.12	4.75	3.50	3.30
	U	2.40	3.25	3.66	6.03	**6.75**	6.50	4.80	3.60	3.45
**Mono.**	F	2.00	2.90	3.30	5.75	**6.32**	6.00	4.50	3.40	3.10
	U	2.02	3.00	3.50	6.00	**6.65**	6.32	4.80	3.45	3.30

F: filtered at the final stage of harvesting (-log CCID_50_/ml)

U: harvested without filtration (-log CCID_50_/ml)

Susp.: cell suspension as substrate

Mono.: cell monolayers as substrate

The obtained measures were evaluated using the T test (paired samples test). Statistical analyses showed that the titre of harvested viruses following inoculation at a MOI of 1:10 has a significant difference in comparison with the other groups, but the difference in viral titre was not significant when the filtration process or considered cell suspensions hired. According to the results, best viral yield was achieved when the seeds were inoculated to MRC-5 cell suspensions with a MOI of 1:10. So, the protocol for preparation of standard seeds was fixed as below:

-Preparation of a suitable amount of MRC-5 cell suspensions in flasks.

-Inoculation of desired seed to cell suspensions with a MOI of 1:10.

-Incubation of the inoculated flasks at 36-37°C for 48 hours.

-Replacement of the media and incubation at 33-34°C for 5-6 days.

-Collection of the harvested viruses in a sterile glass bowl.

-Adding an equal volume of suitable stabilizer.

-Mixing the prepared pool using a magnet stirrer.

-Filtration of the prepared pool using a 0.22 μm Millipore filter.

-Dispensing the filtrate in desired volumes and sampling.

-Quality control test on samples.

As a first step in establishment of the standard seed lot system, a pre-seed virus on passage number 14 was inoculated to MRC-5 cell suspensions. The harvested viruses and their samples were labelled as Master Seed. The master seed was inoculated to MRC-5 cell suspensions with the same method and Mother Seeds were harvested. Then, the mother seed was used as seeds for preparation of working seeds. As the last stage, the working seed was also inoculated to MRC-5 cell suspensions and harvested viruses labelled as vaccines. Quality control tests were carried out at all stages of seed preparation. The properties of harvested viruses were summarized in Table 2.

**Table 2:** Properties of the harvested viruses during preparation of standard seeds

Seed stage	Passage No.	Titre (-log CCID_50_/ml)	Sterility test		Mycoplasma	
			bacterial	fungal	Culture	PCR
Pre-seed	14	3.5	Passed	Passed	Neg.	Neg.
Master seed	15	6.00	Passed	Passed	Neg.	Neg.
Mother seed	16	6.16	Passed	Passed	Neg.	Neg.
Working seed	17	6.20	Passed	Passed	Neg.	Neg.

Several batches of experimental vaccines were produced using prepared seeds. Clinical trials are in progress using these experimental vaccines. On successful completion of the trials we hope to incorporate RS-12 strain in MMR vaccines in place of Hoshino strain in near future.

## Discussion

One of the most important considerations in production and administration of live attenuated viral vaccines is the safety and immunogenicity of the viral strains which are incorporated in these vaccines.

During last several decades, some strains of MuV have been developed for induction of protection against mumps disease in human. These strains differ in their severity of adverse events when administered to the recipients.

RS-12 strain of MuV has been developed at Razi Vaccine and Serum Research Institute. The wild-type virus of this Iranian strain has been isolated in Vero cell culture using a clinical sample, then adapted to human diploid cell culture and finally attenuated by serial passage in the same cell strain. According to previous data, RS-12 is a safe and immunogenic strain with no or slight adverse events in vaccinees which makes it comparable with one of the best recent strains, Jerryl-Lynn. So, it is a good alternative for the vaccine manufacturing companies which are not satisfied with their strain.

As a critical part of the study, the condition of propagation was optimized. Although the viral yield using cell suspension was only slightly higher than monolayer; since the viral propagation using suspension was easier and involves less manipulation on flasks, it was the preferred method of cultivation. The best viral yield was achieved when the seeds were inoculated to cells at MOI of 1:10. At the end of cultivation period, half of harvested materials of each group subjected to filtration. There was no remarkable difference between these two conditions. Despite a small reduction in titre after filtration, the filtration of harvested materials was included in the preferred protocol for preparation of seed lot system. Such a decision has made because the filtration process eliminates any possible residual contaminations.

According to WHO technical reports an important feature of a good viral propagation process is the consistency of the results, primarily the titre. In the case of this study, the process well optimized and there was no fluctuation in the titre of harvested viruses during preparation of seeds (Table 2).

After optimization of the viral propagation, a standard seed lot system was established which was met all WHO recommendations and requirements. All quality control tests were passed successfully. Since RS-12 strain have some advantages in comparison to other strains, the prepared seeds are very valuable. One of the most important advantages of this strain is that the seeds were prepared in MRC-5 (human diploid) cells, which makes it safe for administration to the person with allergic reaction to egg proteins. In addition, using MRC-5 cells as substrate eliminates the risk of contamination of vaccines with poultry viruses that could potentially reach the viral vaccines when chicken embryo fibroblasts are used.

Several experimental batches of MMR vaccine were produced using prepared seeds. Clinical trials were designed based on local and international regulations to complete our data on incorporation of Iranian RS-12 strain in mumps and MMR vaccines. These clinical trials are in progress and the primary results are satisfying.
